# Computational Analysis of SPI1 Missense Mutations and ADMET-Guided Molecular Docking of Cinnamic Acid Targeting the PU.1 ETS Domain: Implications for Hematopoietic Dysregulation and Leukemogenesis

**DOI:** 10.3390/ijms27104278

**Published:** 2026-05-11

**Authors:** Mariam M. Jaddah, Samer N. Khalaf, Mohammed Mukhles Ahmed, Aisha Abdullah Alshanqiti

**Affiliations:** 1 Department of Clinical Laboratory Sciences, College of Applied Medical Sciences, Taibah University, Al Madinah Al Munawarah 42353, Saudi Arabia; mjidah@taibahu.edu.sa; 2 Department of Biotechnology, College of Science, University of Anbar, Ramadi 31001, Iraq; samer.naji@uoanbar.edu.iq; 3 Department of Physiology, College of Medicine, Umm Al-Qura University, P.O. Box 715, Makkah 24382, Saudi Arabia; aamshanqiti@uqu.edu.sa

**Keywords:** SPI1, missense nsSNPs, AML, hematopoiesis, in silico bioinformatics, protein structure, ETS domain, transcription factors, TCGA, Cinnamic acid

## Abstract

Spi-1 Proto-Oncogene (*SPI1*) encodes Purine-rich box 1 Transcription Factor (PU.1), a transcription factor of the ETS family that regulates hematopoietic lineage commitment and immune cell differentiation. Alteration of PU.1 dose or ETS domain integrity may interfere with transcriptional programs, which adds to hematopoietic dysregulation and leukemogenesis. Even though changes in *SPI1* expression have been associated with acute myeloid leukemia (AML), the structural and regulatory effects of missense mutations at the PU.1 ETS domain have not been entirely studied, and targeting the PU.1 ETS domain by ligands is an area of computational analysis that should be further pursued. To computationally describe deleterious missense variants of SPI1 in terms of structural stability, evolutionary conservation, post-translational modification (PTM) context and interaction networks, and to measure ADMET-mediated molecular docking of cinnamic acid with the PU.1 ETS domain (8EQG) as a potential modulator. Missense nsSNPs were obtained through Ensembl and narrowed down by consensus prediction of pathogenicity (PredictSNP, combining SIFT, PolyPhen, SNAP and PhD-SNP and other tools). InterPro/UniProt was used for domain mapping. SWISS-MODEL was used to produce wild-type and mutant PU.1 versions, which were analyzed on the structural alignment and Cα–Cα displacement parameters in UCSF Chimera (v1.19). The estimation of stability change was carried out with I-Mutant and MUpro. Prediction of PTM sites was done using MusiteDeep and exploration of functional partners was done using STRING. Human, mouse and zebrafish orthologue conservation was measured by means of MAFFT alignment. GEPIA2 was used to compare the expression of SPI1 in AML (TCGA-LAML) and normal tissues (GTEx). AutoDock Vina (grid center 6, −2, −9 A; 20 × 20 × 20 A; 16 exhaustiveness) was used to prepare cinnamic acid and dock it into the PU.1 ETS domain (8EQG), with SwissDock being used for consistency checks. SwissADME and ADMETlab 2.0 were used to predict drug-likeness, pharmacokinetics, and toxicity. Nine missense mutations were routinely considered as deleterious with the majority of them being located in or near the ETS DNA-binding domain. Structural comparisons showed local perturbations of the structure and I189F and H211P yielded the greatest conformational changes between prioritized variants whereas other forms had minimal movements. A predominantly destabilizing trend was supported by stability prediction whereby V241G had the strongest destabilization signal with further destabilizations being predicted in I189F and R259C. PTM mapping revealed several potential regulatory residues (phosphorylation, acetylation, ubiquitination, and methylation), which indicated that there could be crosstalk between the sequence variation and the transcriptional regulation. The SPI1 was placed in a central hematopoietic transcriptional module (containing RUNX1, CEBP members, GATA1 and IRF factors) by the STRING network. The cross-species alignment showed that there was high conservation of a number of the mutation sites, which would support functional constraint at the ETS region. The expression analysis revealed that the level of SPI1 mRNA in AML was significantly elevated compared to normal tissues. Docking also indicated a slight and reproducible interaction of cinnamic acid with the ETS domain (top affinity −4.27 kcal/mol), with a solitary leading polar anchor and supportive hydrophobic interactions, which is akin to interaction between fragments. The ADMET profiling revealed the likelihood of success in the oral drug-likeness and low CYP inhibition liability, as well as signifying the presence of a possible hepatotoxicity signal that needs further confirmation through experiments. Comprehensive computational studies suggest that certain pathogenic variants of SPI1 missense defects, especially in the ETS domain, can result in loss of PU.1 structural stability and regulatory environment, which are in line with the disturbed hematopoietic regulation and AML-related dysregulation. Cinnamic acid demonstrates moderate yet reproducible binding to the PU.1 ETS domain and has an overall favorable developability profile, which indicates that it is better considered as a starting scaffold, as opposed to an active inhibitor. The results give a logical basis of focused biochemical validation and structure-directed optimization of ETS domain modulators in hematologic disease settings.

## 1. Introduction

Transcription factors (TFs) play an essential role in regulating gene expression, controlling cellular fate, proliferation, differentiation and apoptosis. One of the key TFs that contributes to hematopoiesis is *SPI1*, which encodes for the ETS domain transcription factor PU.1. PU.1 is critical for the specification, differentiation and maturation of different blood cells lineages, including macrophage, B-cells, monocytes, granulocytes and dendritic cells [1]. PU.1 binds to purine-rich DNA motifs to regulate transcriptional programs and recruit co-factors to control chromatin accessibility during immune cell development [2].

Dysregulation of SPI1 has been associated with the pathogenesis of hematologic malignancies, especially myelodysplastic syndromes (MDS) and acute myeloid leukemia (AML) [3]. Overexpression of PU.1 can disrupt normal hematopoietic homeostasis, while a decrease in its expression levels leads to block differentiation and promotes leukemic stem cell self-renewal [4]. This dosage sensitivity shows the importance of SPI1 to maintain the normal hematopoiesis development.

Although several functional and structural domains of SPI1 have been well-examined, including the C-terminal ETS DNA-binding domain, how particular missense variants (nsSNPs) change its structure and function remains unexplored. Missense mutations can alter protein folding, stability, and interaction surfaces, thereby influencing TF activity. Given the SPI1 role in hematopoietic balance, investigating the consequence of these mutations is crucial for understanding cancer progression and congenital immunodeficiency.

Recent advances in computational bioinformatics nowadays provide large-scale functional assessment of missense variants. Predicting the impact of amino acid alterations on protein stability and function can be performed via algorithms such as MutationAssessor, PolyPhen-2, STFT and I-Mutant 3.0. Other tools such as Phyre2 and SWISS-MODEL, along with visualization software such as ChimeraX (v1.19), allow valuable insights into how these missense mutations can impact on protein conformation, particularly in the conserved domains. Post-translational modification (PTM) prediction tools such as ModPred and MusiteDeep reveal how variants interact with regulatory mechanisms such as methylation, acetylation, ubiquitination and phosphorylation. All of these processes are associated with SPI1 function during leukemogenesis and immune response [5,6,7].

Evolutionary conservation highlights functionally residues. Highly conserved residues across species such as human, zebrafish and mouse reveal essential biological functions. For instance, zebrafish (Danio rerio) is considered as an excellent model organism to study hematopoiesis during embryogenesis due to ease of genetic manipulating, optical transparency and conserved genetic pathways [8]. Therefore, examining *SPI1* mutations in different species enriches our understanding about their developmental and evolutionary relevance.

Furthermore, as PU.1 does not perform its function in isolation, examining SPI1 protein–protein interactions (PPI) network is essential. It forms network cooperation with hematopoietic transcriptional regulators such as CRBPA, GATA1, RUNX1 and IRF4/8, all of which are crucial for immune response and lineage specification [4,9,10]. Therefore, structural mutations may disrupt these interactions, which may then alter cell fate pathways or impact immune competence.

Transcriptional data from The Cancer Genome Atlas (TCGA) has shown abnormality in SPI1 expression in leukemia. SPI1 has a role as a tumor suppressor; thus, any increase in its expression may indicate oncogenic transcriptional reprograming [5]. Though the increase in SPI1’s clinical investigations, comprehensive deep examinations of its missense mutations using in silico approaches are still limited. The focus in most of the existing studies was usually on the expression pattern or large deletion, rather than the alterations in the amino acids which could change the protein function.

Cinnamic acid was selected as a fragment-like scaffold with known biological activity and favorable drug-like properties, intended for exploratory binding assessment rather than as a validated inhibitor [11]. Cinnamic acid (trans-3-4-phenyl-2-propenoic acid) is a natural phenylpropanoid derived out of L-phenylalanine using phenylalanine ammonia-lyase and is a major intermediate in plant secondary metabolism [11]. This is due to its structure which comprises a conjugated aromatic ring coupled with an alpha–beta unsaturated carboxylic acid group that provides it with redox activity and hydrogen bonding to biological targets. It has been demonstrated that cinnamic acid and its derivatives have antioxidant and anti-inflammatory effects, in part by the regulation of oxidative stress signaling and inflammatory mediators [12]. Also, cinnamic acid analogs show antimicrobial action against different strains of bacteria; furthermore, in antiproliferative cancer-related research, the phenylpropanoid skeleton is an attractive lead structure in medicinal chemistry optimization [13]. Cinnamic acid is drug-like in terms of having a rule of five compliance with Lipinski and physicochemical characteristics that are supportive of oral bioavailability [14]. The cinnamic acid is an appropriate fragment-like scaffold due to its low molecular weight, and it possesses a potential modification framework that can be explored to design drugs and through computer-aided discovery.

Cinnamic acid was selected in this study as a fragment-like phenylpropanoid scaffold with favorable drug-like properties rather than as a pre-validated inhibitor of the PU.1 ETS domain. Its structural simplicity and documented biological activity make it a suitable candidate for exploratory docking, aimed at assessing initial structural compatibility with the ETS DNA-binding interface and providing a basis for future scaffold optimization. Consequently, a seamless combination of (i) computational evaluation of SPI1 missense mutants with (ii) structural examination of the PU.1 ETS domain and (iii) ADMET-directed docking of cinnamic acid can provide a sensible framework to relate sequence variation, structural stability and ligandability hypotheses pertaining to hematopoietic malregulation. This paper implements such a combined in silico approach to rank potentially toxic SPI1 replacement, decode their site of location in conserved functional domains, and consider the potential of cinnamic acid binding to the PU.1 ETS domain as a starting point towards subsequent experimental validation and rational optimization. 

## 2. Results

### 2.1. Identification of Deleterious nsSNPs in the SPI1

In this section, nsSNPs that may interfere with the function or structure of SPI1 protein in humans were identified. To achieve this, several in silico prediction tools were used including PredictSNP, SIFT, MAPP, PolyPhen1, PolyPhen2, and PhD-SNP. Among 825 nsSNPs examined, 9 nsSNPs were consistently predicted to be deleterious across all tools employed (Table 1).

### 2.2. Most of Identified nsSNPs Are Located Within the Functional Domain of SPI1

**Figure 1:**
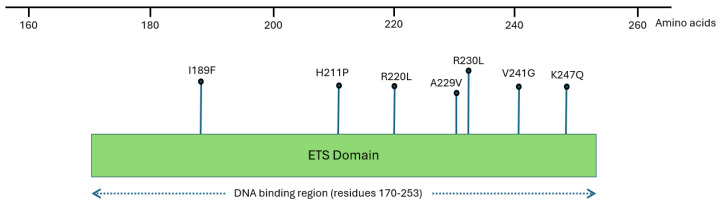
Domain mapping of SPI1 protein using InterPro server. This figure illustrates that seven out of nine missense mutations of interest are situated within the ETS domain of the SPI1 protein, particularly within the DNA-binding region (residues 170–53). This figure was generated based on the information of InterPro server and UniProt (DI: P17947).

All the identified nine variants are located within or near the DNA-binding domain of the ETS domain of SPI1, specifically within the winged binding DNA motif, as shown via InterPro analysis (Figure 1). This domain is crucial for SPI1 role as a transcription factor regulating hematopoietic cell fate. Thus, mutations within the DNA-binding domain might impact protein folding, which might cause a defect in hematopoietic cell differentiation.

### 2.3. Structural Evaluation of WT and Mutant SPI1 Proteins

**Figure 2:**
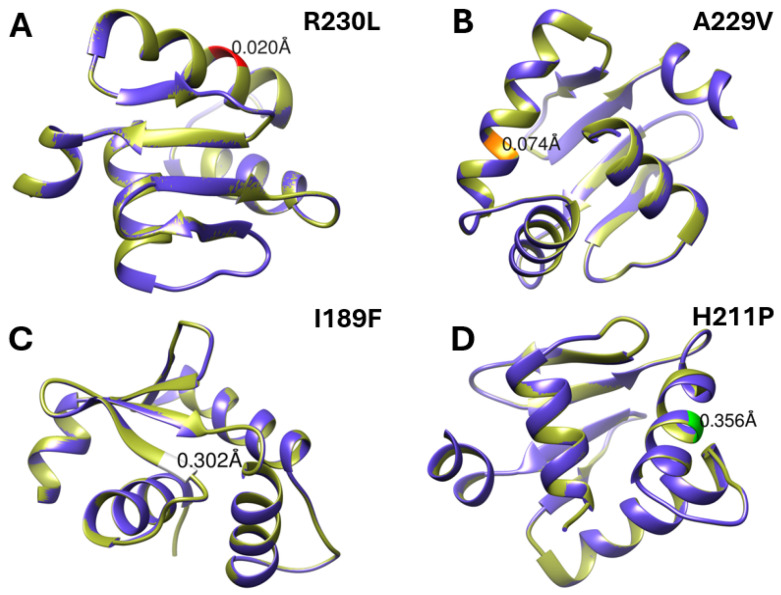
Structural comparison of wild-type and mutant SPI1 within the ETS DNA-binding domain. Structural modeling and visualization were restricted to the ETS domain to ensure structural reliability and avoid artifacts associated with intrinsically disordered regions. (**A**) R230L, (**B**) A229V, (**C**) I189F, and (**D**) H211P variants are shown, with mutation sites highlighted. The structures demonstrate varying degrees of local conformational deviation, with I189F and H211P exhibiting comparatively greater structural perturbations, while R230L and A229V display minimal structural changes.

Structural comparisons were restricted to the ETS DNA-binding domain to avoid artifacts associated with modeling intrinsically disordered regions of SPI1 and to ensure biologically meaningful interpretation. Structural alignment of WT-SPI1 with each mutation showed localized confirmational variations at the mutation positions. The R230L mutation, located within the ETS DNA-binding domain, revealed a slight structural change. The Cα–Cα distance between the WT and mutant residue was only 0.020 Å (Figure 2A). Likewise, the Cα–Cα distance between WT and A229V residue, located in the same domain, was slightly more at 0.074 Å (Figure 2B). Interestingly, the I189F and H211P displayed more substantial structure alteration, with Cα–Cα distances of 0.302 Å and 0.365 Å, respectively (Figure 2C,D). The conformational alteration in H211P may be attributed to introduce of proline, a known helix-disrupting residue, which may affect the secondary structure within the ETS domain. The Cα–Cα distance between the wild-type and the other five missense mutant residues ranged from 0.010 Å to 0.060 Å (Figure 2. These results suggest although all the four mutations impact the ETS domain, I189F and H211P displayed a significant structural impact on structural stabilization of SPI1 protein.

### 2.4. Protein Stability Prediction for SPI1 Mutations

The protein stability analysis revealed 7 out of 9 missense variations were predicted to decrease protein stability. The most pronounced destabilization was observed for the V241G mutation in both I-Mutant and MUpro with ΔΔG = −3.35 kcal/mol and −2.41 kcal/mol, respectively (Table 2), indicating a huge structural impact. Similarly, both I189F and R259C mutations displayed prediction with significant impact on protein stability. H211P variant showed a partial discrepancy between tools. The protein stability was increased slightly as shown by I-Mutant (−0.70 kcal/mol) while strong destabilization was predicted via MUpro (−1.50). In contrast, R230L was predicted to be stabilizing via MUpro but destabilizing via I- Mutant. Combining the prediction by the two tools showed more validated and robust insight for the pathogenicity of these variants, particularly for those that are situated within the ETS domain.

### 2.5. Determination of SPI1 Post-Translational Modification Sites

Multiple residues within the SPI1 protein were predicted to be sites for post-translational modifications (PTM) (Table 3). Three phosphorylation sites were detected at threonine 164 and serine residues 166 and 188, with scores of 0.192, 0.379 and 0.177, respectively. These modifications may play a role in transcriptional activity and signal transduction.

One ubiquitination site and three acetylation sites were identified at lysine residues 221, 242, 246 and 247, suggesting their involvement in chromatin remodeling or protein stability. Among the three sites, lysine 242 exhibited the highest score at 0.413, while lysine 221 for ubiquitination showed a score at 0.336. Additionally, four arginine residues (212, 220, 230 and 259) were predicted as methylation, recording the highest score at 259 residues at score 0.93 and indicating potential regulatory function. Finally, a glycosylation site was predicted at asparagine 219 with a score at 0.033. These findings show several sites that are predicted to contribute to modulate SPI1 via different post-translational process. The modifications may influence SPI1 protein activity by affecting chromatin remodeling, signal transduction and transcriptional process.

### 2.6. Interaction Network of SPI1 Protein

**Figure 3:**
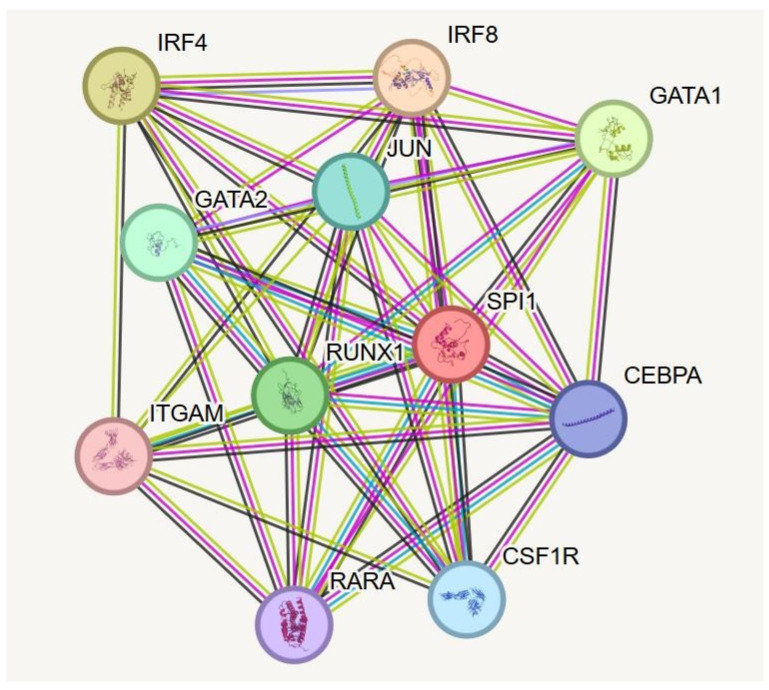
SPI1 interaction network of SPI1 protein. The network illustrated an interaction between SPI1 and some key regulators of immune system, hematopoiesis and transcriptional factors control. This figure was generated by the STRING database.

The analysis showed a direct functional association between SPI1 and several key regulators of immune cell development, hematopoiesis and transcriptional control (Figure 3). Notably, SPI1 directly interacts with GATA1, a transcription factor involved in regulation of B-cell developments. RUNX1, a crucial regulator for hematopoietic lineage commitment, also showed a direct interaction with SPI1. Additionally, CEBPA and CEBPB, transcription factors essential for monocytes and granulocytes differentiation, were present in the interaction network. Finally, IRF4 and IRF8, which are transcription factors important for dendritic cells and myeloid differentiation, were detected in the interaction network of SPI1. Table 1 presents information about the predicted functional partners of SPI1.

### 2.7. Conservation Analysis of Missense Mutations

**Figure 4:**
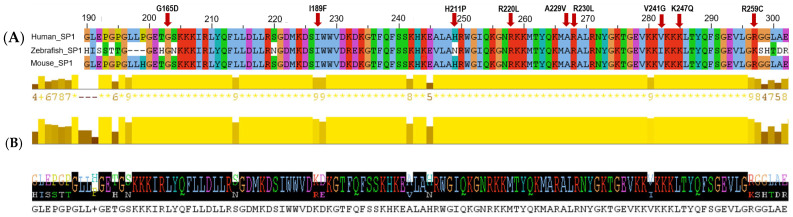
Multiple sequence alignment and conservation analysis of SPI1. (**A**) Multiple sequence alignment of SPI1 orthologs highlighting conserved residues within the ETS domain, with mutation sites indicated by red arrows. (**B**) Conservation and quality profiles derived from the alignment, along with the consensus sequence, illustrating regions of high evolutionary conservation.

A multiple sequence alignment (MSA) for SPI1 protein sequences for human, zebrafish and mouse was performed to detect the evolutionary conservation of the nine missense mutations (Figure 4). In all three species analyzed, residues G165, I189, R220, A229V, R230, and K247 are displayed to be highly conserved as illustrated by the tall conservation bars in yellow color. In contrast, H211, V241 and R259 residues displayed more variability, especially in zebrafish sequences, and are shown as shorter conservation yellow bars. The findings revealed that most of the variation sites, particularly those located within the ETS domain, are highly conserved among the three species, highlighting their functional importance for transcriptional activities and DNA-binding.

### 2.8. Upregulation of SPI1 Expression in Acute Myeloid Leukemia (LAML)

**Figure 5:**
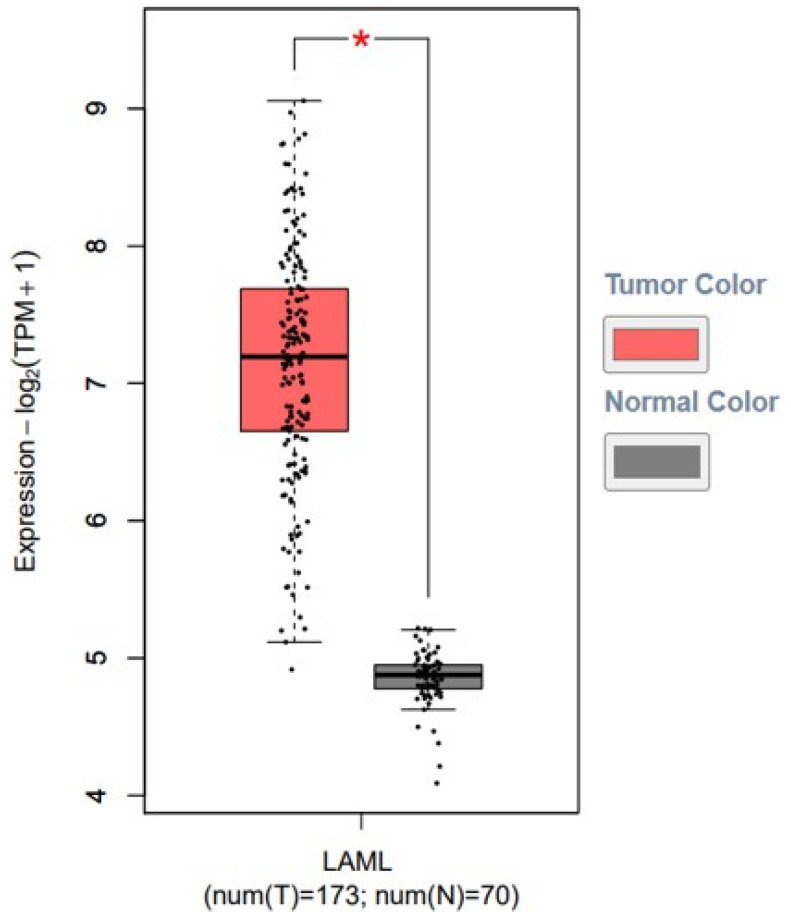
Differential expression of SPI1 between LAML tumor and normal tissues based on TCGA data via GEPIA2. The bar plot revealed a greater expression level of SPI1 in tumor tissue (pink, n-173) compared to normal tissue (gray, *n* = 70). Expression values are illustrated as log_2_ (TPM + 1). A red asterisk refers to statistical significance (*p* < 0.01). * = Statistical Significance.

Analysis of TCGA data showed that SPI1 expression was significantly upregulated in LAML tumor tissue compared to normal tissue (Figure 5). The median expression levels were approximately log_2_ (TPM + 1) = 7.0 and 5.0, respectively. The difference was statistically significant (*p* < 0.01), suggesting possible transcriptional dysregulation of SPI1 in leukemia.

### 2.9. Molecular Docking Analysis of Cinnamic Acid Against 8eqg_modified.pdb

**Figure 6:**
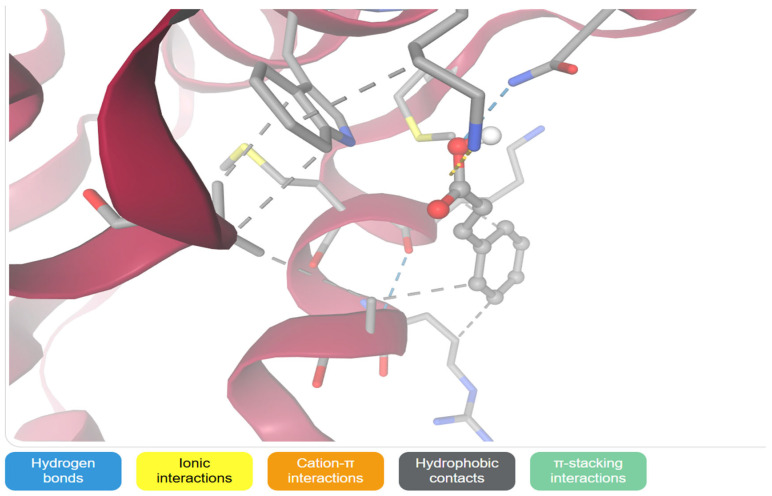
3D interaction between cinnamic acid with 8EQG. The cinnamic acid establishes several favourable interactions such as hydrogen bonding, ionic bonds, cation-π bonds, hydrophobic bonds, and π-stacking with neighbouring amino acid residues. All of these interactions help to stabilize and enhance binding of the protein to the ligand. The blue dashed lines indicate hydrogen bonding, the yellow dashed lines indicate ionic interactions, the orange dashed lines indicate cation–π interactions, the gray dashed lines indicate hydrophobic contacts and the green dashed lines indicate π-stacking interactions.

Figure 6 shows AutoDock Vina resulting in the docking of cinnamic acid (C1=CC=C(C=C1)/C=C/C(=O) O) into the binding site of 8eqg_modified.pdb produced twenty ranked binding conformations with affinity scores between −4.270 and −3.421 kcal/mol. The pose with the highest score (Model 1) had a binding energy of −4.270 kcal/mol with small differences in energy between the top-ranking poses (0.3 kcal/mol between the first three poses). The occurrence of this narrow distribution shows an easy and repeatable binding orientation within the defined docking grid (20 × 20 × 20 A; exhaustiveness 16) as opposed to a population of separate and energetically distinct binding modes.

The binding affinity calculated represents small thermodynamic stabilization of the protein–ligand complex. Energies in this regime are generally weak to moderate non-covalent interactions which are largely governed by van der Waals forces and weak hydrogen bonding as opposed to strong electrostatic anchoring or strong catalytic interactions. The lack of poses with energies significantly lower indicates that cinnamic acid does not bind to highly optimized or deep active-site cavity but rather binds to a fairly shallow binding site.

Structurally, cinnamic acid is a rigid and planar molecule that consists of an aromatic phenyl ring and is conjugated by a trans-alkene functional group on one end of the molecule to a carboxylic acid group. The carboxylic acid moiety seems to be the main center of polar interactions that has one or two directional hydrogen bonds with adjacent polar amino acid residues that line the pocket. The extent of the interaction network is however limited and there is no indication of strong salt bridge formation or multi-point hydrogen bonding which is why the binding energy is moderate.

The aromatic ring is also involved in stabilization through the hydrophobic interactions as well as the possible π interactions and cation interactions with other residues. With these interactions, enthalpic stabilization is promoted but is not very strong because the molecular surface area and the exposure of the solvent to the ligand is limited. The conjugated alkene spacer is rigid and electronically delocalized but structural rigidity limits conformational flexibility so that optimum steric complementarity between irregular pocket geometries cannot be achieved.

Throughout the docking ensemble, there is little difference in binding energies, which suggests that cinnamic acid assumes a stable preferred orientation that is determined by its planar scaffold. Nevertheless, the interaction pattern is dominated by scarce hydrogen bonding and moderate hydrophobic packing but there is no indication of profound occupation of catalytic sites and interwoven interaction patterns.

Generally, the outcomes of the docking process indicate that cinnamic acid is structurally plausible, but loosely complexed with 8eqg_modified.pdb. Although the reaction is regular and thermodynamically favorable, the small binding affinity indicates that cinnamic acid would be better used as a scaffold fragment in place of an effective inhibitor. Probable structural alteration or functional group enlargement would be necessary in order to increase interaction density, binding strength, and possible biological efficacy.

### 2.10. Molecular Binding Interaction Analysis of Cinnamic Acid with 8EQG

**Figure 7:**
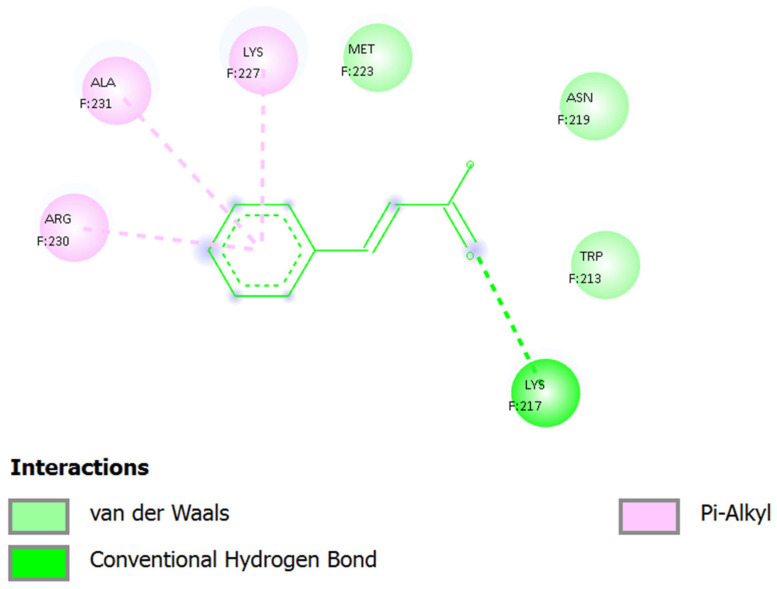
2D interaction between cinnamic acid with 8EQG. The ligand showed typical H-bonding with Lys217, π–alkyl with Ala231, Lys227 and several van der Waals contacts with other residues around the binding site, such as Trp213, Asn219, Met223 and Arg230, which are responsible for stabilization of the protein–ligand complex within the binding pocket.

The interaction of Cinnamic Acid within the binding pocket of 8EQG is shown in Figure 7, which is structurally stable yet moderately weak, with limited number of polar interactions and major number of hydrophobic interactions with the 8EQG binding pocket. The ligand takes on its typical planar structure, which is determined by the conjugated phenyl alkenecarboxylic acid backbone, and fits into a shallow cavity, as defined by both polar and non-polar residues.

The major stabilizing interaction is a conventional hydrogen bond between the carboxylic acid group of Cinnamic Acid and the protonated ε-amino group of Lys217, with the carbonyl oxygen serving as a hydrogen bond acceptor to the protonated ε-amino group of the lysine residue. This interaction may be further strengthened by partial electrostatic attraction because Lys217 is positively charged in physiological conditions. This hydrogen bond serves as the main anchoring site; the ligand is positioned in the pocket and unnecessary rotational flexibility is limited. Nevertheless, there are no more powerful hydrogen bonds or the formation of a salt bridge, which restricts the overall binding strength.

Around the aromatic ring, there are Ala231, Lys227 and Arg230, which provide π–alkyl interactions which stabilize the phenyl system with good interactions between the π–electron cloud and the aliphatic parts of their side chains. The interactions confer hydrophobic confinement and hold the spatial positions of the aromatic moiety. Each of these interactions are weak in isolation compared to classical hydrogen bonds; however, when combined, they collectively enhance enthalpic stabilization and minimize solvent exposure.

Other residues such as Met223, Asn219, and Trp213 are involved in van der Waals interactions, indicating a high level of steric complementarity but not strong directional interactions. The flexible thioether side chain on Met223 is probably optimized so as to maximize packing into the surface, and the hydrophobic or edge-to-face aromatic interaction of Trp213 may be involved. Asn219 is a polar amino acid that might affect the stabilization of the local microenvironment although it is not shown to form a strong hydrogen bond with the ligand.

Generally, cinnamic acid fits in an outer part of the binding cavity other than penetrating a catalytic core. A single polar anchor and surrounding hydrophobic packing prevails in the interaction network, which leads to modest stabilization in accordance with the docking affinity (=−4.27 kcal/mol). The low molecular weight and inflexible planar shape restrict the number of points of interaction and cannot occupy a pocket extensively. As a result, cinnamic acid acts like a fragment-like binder, forming a structurally plausible, but energetically moderate, complex with 8EQG. Strengthening of the binding density and increased inhibitory capacity would probably require structural extension or modification of functional groups.

### 2.11. Pharmacokinetic and Toxicological Characterization of Cinnamic Acid

**Figure 8:**
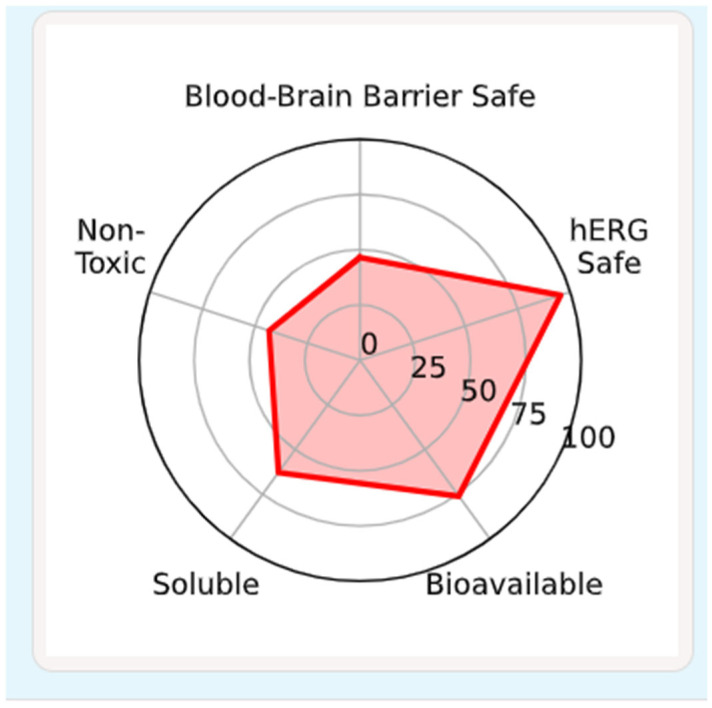
ADMET of cinnamic acid. The results show that cinnamic acid has desirable features in terms of bioavailability, hERG safety, aqueous solubility, blood–brain barrier safety, and non-toxicity, which suggest potential drug-likeness and pharmacological suitability as a bioactive drug candidate.

Table 4 and Figure 8 show the cinnamic acid (MW = 148.16 Da) has a tight molecular structure that highly favors passive diffusion across the membrane. It has low molecular weight with a LogP of 1.78 which places it in the best physicochemical window of oral small-molecule therapeutics. In terms of hydrophilicity versus lipophilicity, the balance is an indication of good partitions across biological membranes without being over-accumulated in lipid-rich regions. High permeability is further promoted by the presence of one hydrogen bond donor and one hydrogen bond acceptor along with a topological polar surface area (TPSA) of 37.30 A 2. A TPSA below 70 A 2 is usually characteristic of good intestinal absorption and possible blood–brain barrier (BBB) penetration; therefore, cinnamic acid meets the conditions theoretically. Its drug-like property (quantitative estimate, QED = 0.65) supports the fact that it is a lead-like molecule, but not a structurally complicated drug candidate.

In terms of absorption, the intestinal absorption (0.99) and oral bioavailability (0.91) are predicted with almost 100 percent uptake after the drug was administered orally. These are in harmony with its moderate aqueous solubility (−2.36 log mol/L) which indicates that it can dissolve adequately in gastrointestinal fluids and remain permeable across the membrane. The estimated cell permeability (−4.58 log10^−6^/s) confirms that there is efficient passive diffusion. Notably, a low P-glycoprotein (P-gp) inhibition (0.004) shows that there is minimal interference with efflux transporters, which decreases chances of drug–drug interactions between transporters.

BBB is moderate in terms of central nervous system accessibility, given its penetration probability (0.77), suggesting that it has average accessibility, which can be beneficial or not based on the therapeutic intent. The high plasma protein binding (92.47) indicates that there is a minimal amount of free fraction in circulation, although high binding might increase apparent exposure and rapid clearance. The calculated volume of distribution (4.61 L/kg) shows the distribution into the tissues reaches far into the extravascular space, which is in line with its lipophilic nature.

Cinnamic acid has a desirable cytochrome P450 interaction profile of a metabolic nature. This low-predicted inhibition of major isoforms (CYP1A2, CYP2C19, CYP2C9, CYP2D6, and CYP3A4) is an indicator of a low risk of developing all-encompassing metabolic drug–drug interactions. The average potential of CYP2C9 substrate action (0.44) indicates that hepatic biotransformation could be done by this route. The microsomal clearance (3.22 µL/min/mg) is found to be in support of moderate metabolic turnover, but due to the fact that the predicted half-life is 0 h, it is evident that it is ejected by the system quickly; this is possibly through efficient conjugation of the system by the liver and excretion by the kidneys.

The toxicological data is usually also reassuring with regard to cardiotoxicity (hERG inhibition 0.01), in addition to mutagenicity (0.02) being low-risk. Acute toxicity is abated on the basis of LD50 forecasts. Nevertheless, the probability of liver injury as a result of drugs is high (0.91) and should be considered. Seeing that cinnamic acid is hepatically metabolized, such an indication could indicate possible metabolic stress or the formation of reactive intermediates. The moderate potential of skin irritation (0.61) is in line with the moderate irritant effects at elevated levels.

All in all, cinnamic acid has an excellent oral absorption, widespread tissue distribution, clearance, low interaction liability, and low acute toxicity. The main issue that arises out of this in silico analysis is the fact that it predicts a high risk of hepatotoxicity; this would have to be carefully confirmed by conducting experimental toxicological analyses.

### 2.12. Swiss Target Prediction Profile of Cinnamic Acid

**Figure 9:**
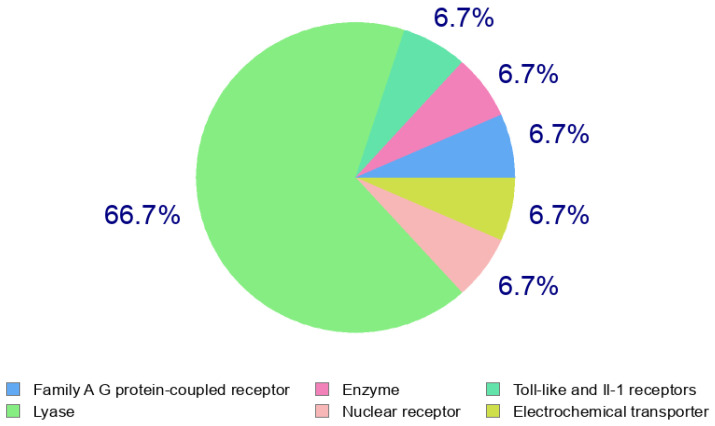
Swiss Target Prediction Profile of Cinnamic Acid.

The Swiss Target Prediction analysis of cinnamic acid provided a multi-target interaction pattern involving G protein-coupled receptors (GPCRs), metabolic enzymes, ion channels, nuclear receptors, transporters, proteases, phosphatases and kinases (Table 5 and Figure 9). Hydroxycarboxylic acid receptor 2 (HCAR2, UniProt: Q8TDS4) showed the highest probability score of 0.8870, which is significant, therefore indicating a high probability of interaction. HCAR2 is a Class A GPCR which plays a role in lipid metabolism, anti-inflammatory signaling and immune modulation. This receptor is highly expressed, indicating that cinnamic acid could have metabolic and immunoregulatory actions via the pathways of GPCR.

One enzymatic target, Aldose reductases (AKR1B1, P15121), had an average probability (0.1012) of being targeted. Aldose reductase is an important oxidoreductase that plays an important role in the polyol pathway and diabetic complications. Its forecast is in line with other past reports which characterize the presence of antioxidant and metabolic regulatory functions of phenylpropanoid derivatives.

Components of inflammatory signaling, such as the homologous Toll-like receptor 4 (TLR4, O00206), were also predicted to be interfered with (0.0918), implying that it may interfere with innate immune activation and NF-KB-mediated signaling.

Three isoforms of carbonic anhydrase were always predicted, which include carbonic anhydrase II (CA2, P00918), carbonic anhydrase I (CA1, P00915), and carbonic anhydrase IX (CA9, Q16790) (probability ranges 0.0823–0.0727). These lyases control homeostasis of pH and they are often linked to adaptation of microenvironment of tumors, especially CA9 in hypoxic tumors. Their existence indicates potential functions in acid base regulation and metabolic reprogramming in cancer.

Estrogen receptor beta (ESR2, Q92731) suggested the role of the nuclear receptor in the potential of endocrine-modulatory activity. Monocarboxylate transporter 1 (SLC16A1, P53985) and transient receptor potential cation channel A1 (TRPA1, O75762) depicted transport and sensory pathways, respectively, and it was possible that they were involved in metabolic flux and nociceptive signaling.

The enzymes of extracellular matrix remodeling, including matrix metalloproteinase 9 (MMP9, P14780) and matrix metalloproteinase 2 (MMP2, P08253), were predicted with medium probabilities (0.0535), suggesting the possibility of anti-invasive or anti-metastatic relevance. Also, intracellular signaling modulators (protein–tyrosine phosphatase 1B, PTPN1, P18031) and epidermal growth factor receptor (EGFR, P00533) were found, which is indicative of a potential adjustment of proliferative and metabolic processes.

The combination of the predicted targets shows that cinnamic acid has a pleiotropic pharmacological profile. Its prevalence of GPCR signaling, and anti-inflammatory and proliferative pathways, contribute to the description of this multi-functional approach to bioactive scaffold with metabolic, anti-inflammatory and possible anticancer implications.

## 3. Discussion

SPI1, also known as PU.1, is a member of the ETS family of transcription factors and is located on chromosome 11 in the human genome. It has crucial roles in hematopoiesis, being highly expressed in myeloid cells, lymphocytes and hematopoietic stem cells [4,15]. SPI1 positively regulates multiple genes in granulocytes/monocytes with the highest levels of expression and B-cells with lower or moderate levels.

Additionally, it is expressed in erythroid precursors and across different hematopoietic progenitors with a potential to develop into lymphocytes lineages [16,17,18]. SPI-deficient mice showed its pivotal role during hematopoietic lineage commitment, demonstrating a defect in the development of lymphocytes, T cells, B cells and other types of immune cells [4,15,19]. The current study was performed to identify and characterize missense nonsynonymous single nucleotide polymorphisms (nsSNPs) within the *SPI1* gene. A comprehensive computational analysis was performed to explore the impact of these mutations on protein structure, stability and function. Additionally, the current work investigates the protein–protein interaction (PPI) network of SPI1 within the hematopoiesis process and its relevance to hematological diseases related to this gene.

This study identified 9 potentially pathogenic missense variants out of 825 nsSNPs in the *SPI1* gene, as predicted by the PredictSNP tool. Seven of these nine variants were found to be located within the ETS domain, while the other two were located close to the region, as determined by InterPro database. The ETS domain consists of 85 amino acids located near C- terminus of the SPI1 protein. It is a highly conserved DNA-binding domain that particularly recognizes the sequence 5′-GGAA/T-3′. It is essential for SPI1 as a regulatory transcription factor [20].

Structural alignment through SWISS-MODEL UCSF Chimera between wild-type and SPI1 protein variants revealed that several missense mutations, although located within the conserved ETS domain, display differential impacts on the conformational integrity of the protein. Among the nine deleterious mutations, structural modeling indicated that variants such as R230L and A229V generated minor deviations, with Cα–Cα displacement of 0.020 Å and 0.074 Å, respectively. These minimal alterations suggest a limited disruption on protein architecture and stability, likely retaining the DNA-binding activity. In contrast, I189F and H211F displayed more shifting in the protein conformation with distances of 0.302 Å and 0.365 Å, respectively. These findings indicate a potential impact on the tertiary structure of SPI1 protein. In particular, H211P may disrupt protein stability due to the conformational rigidity of proline, a residue known to distribute α-helical structures, especially in the TFs domains such as ETS [21]. The I189F variant, while involved in hydrophobic substitution, introduces a bulkier phenylalanine side chain, which may disrupt packing interactions within the ETS domain, suggesting the impairment of the SPI1 protein function.

Collectively, although all the nine mutations are supposed to be deleterious, only the I189F and H211P variants are likely to cause a marked impact on SPI1 structure, stability and function. These two mutations are likely to dysregulate gene expression and contribute to the impairment of hematopoietic development.

In silico prediction using I-Mutant and MUpro revealed that seven out of the nine missense nsSNPs mutations are likely to decrease SPI1 protein stability, leading to DNA-binding activity potentially being affected. Among these, the V241G substitution was suggested to be the most destabilizing for SPI1 protein, with ΔΔG values of −3.35 kcal/mol (I-Mutant) and −2.41 kcal/mol (MUpro); such a reduction in free energy suggests a marked change in the protein-folding environment [22,23]. Similarly, both I189F and R259C mutations were also associated with a reduction in protein stability. The I189F, located within the ETS domain, changes the non-polar isoleucine to an aromatic phenylalanine, disrupting hydrophobic packing in the ETS domain [24]. On the other hand, R259C mutation, though located outside the ETS domain, replaces a positively charged arginine with thiol-containing cysteine, potentially introducing disulfide bridges and altering electrostatic interactions, affecting the structural integrity.

Interestingly, prediction discrepancy between the two prediction tools was shown in the H211P substitution with a slight increase in stability in I-Mutant (−0.70 kcal/mol) and marked destabilization in MUpro (−1.50 kcal/mol). This inconsistency is common in the computational predictions, showing the importance of using multiple algorithms [25]. Given that the α-helices of protein structure may be disrupted via proline residues through the introduction of conformational links, especially in the ETS domains [26], H211P is suggested to cause protein destabilization. Furthermore, R230L mutation also exhibited discrepant prediction, indicating destabilization in I-Mutant and stabilization in MUpro. The substitution of polar arginine-to-hydrophobic leucine may alter the intra-domain interactions and affect solvent exposure [27]. Although the R230L substitution is suggested to cause a mild stability change, given that it is situated in the ETS domain, this might translate to marked functional impacts. Overall, the findings showed that most of the variants, especially those that are located in the ETS domain, affected the SPI1 protein stability, which in turn may affect the lineage commitment, causing immune dysregulation and hematological diseases.

Post-translational modifications (PTMs) are crucial for controlling transcription factors such as SPI1, affecting their localization, stability, and transcriptional activity. A prediction from MusiteDeep determined phosphorylation locations at residues T164, S166 and S188, which may modulate the SPI1 function in lineage-specific regulation and signal transduction. These results are consistent with findings regarding phosphorylation regulation in PU.1 activity during hematopoiesis [28]. Additionally, the predicted methylation at arginine residues 212, 220, 230 and 259 displays a possible regulatory function due to the role of arginine methylation in gene expression and TF interactions [29]. Ubiquitination at lysine 221, alongside acetylation at lysine 142, 246 and 247, predicts a role in the remodeling of SPI1 protein. Ubiquitination regulates protein degradation, while DNA-binding and transcriptional activities are enhanced via acetylation [30,31].

The protein–protein interaction (PPI) of SPI1 revealed a direct network between this protein and several hematopoietic transcription factors, indicating its critical role during hematopoietic regulation. It demonstrates a direct interaction with RUNX1, which is crucial for HSCs generation and differentiation. Previous studies showed cooperation between SPI1 and RUNX1 in hematopoietic gene expression [18]. SPI1 was also shown to interact with GATA1, an essential regulator for lineage fate decision between erythroid and myeloid lineages [32]; additionally, CEBPB, a key regulator for monocytes and granulocytes differentiation [33], was also shown to interact directly with SPI1. Finally, SPI1 also displayed an interaction with IRF4 and IRF8, essential for macrophage and dendritic cell differentiation, suggesting the direct role of SPI1 in immune differentiation [34]. Overall, these interactions highlight the critical role of PU.1 during hematopoietic lineage commitment and immune cell differentiation and specification.

Cross-species conservation analysis among human, zebrafish and mouse genomes showed that the majority of the pathogenic variants, in particular G165, I189, R220, A229, R130 and K247, are highly conserved. Their locations within the ETS domain highlighted their functional importance [35]. In contrast, H211, V241 and R259 showed less conservation in zebrafish, illustrating specific adaptation or the more flexible function of SPI1 in non-mammalian species.

The analysis showed that SPI1 is upregulated significantly in tissues with LAML compared to the normal controls, indicating potential roles during leukemogenesis. This transcription factor has been shown to regulate lymphoid and myeloid lineage differentiation [1]. A dysregulation in SPI1 expression may block the differentiation and proliferation shown in LAML [3]. Thus, the transcriptional reprogramming of LMAL may be the cause of the observed increase in the SPI1 expression in LMAL tissues from TCGA, suggesting the potential to use SPI1 as a prognostic biomarker or therapeutic target in myeloid malignancies.

Cinnamic acid has been biologically reported to have antioxidant and anti-inflammatory effects, which take place, in part, by regulating redox-sensitive signaling pathways and inflammatory mediators [12]. The mechanisms can have an indirect impact on transcriptional regulation in inflammatory or malignant processes. Moreover, the derivatives of cinnamic acids have been shown to have antimicrobial and antiproliferative effects, which confirms the pharmacological significance of the phenylpropanoid backbone [13,14]. Taken together, the current results are consistent with the available literature suggesting that cinnamic acid represents a bioactive low-complexity scaffold with reasonably good pharmacokinetic properties but poor inherent binding affinity and, hence, it should be optimally structured to achieve better therapeutic outcomes. Docking scores reported in this study represent relative interaction energies and should not be interpreted as experimental binding affinities.

The limitations of this study are as follows:

1. The study is entirely in silico, lacking experimental validation (e.g., biochemical or cellular assays).
2. Absence of positive and negative control ligands, limiting the interpretability of docking scores.
3. Docking energies represent approximate interaction scores, not true binding affinities.
4. A single ligand (cinnamic acid) was evaluated without comparative ligand benchmarking or a screening library.
5. Limited exploration of protein flexibility (static docking without extended conformational sampling).
6. Predicted ADMET profiles are computational estimations and require experimental confirmation.

Future studies should therefore do the following:

1. Perform in vitro validation (e.g., binding assays, reporter gene assays for PU.1 activity).
2. Conduct molecular dynamics simulations to assess binding stability and protein flexibility.
3. Include reference ligands (controls) for more robust docking validation.
4. Expand to virtual screening of cinnamic acid derivatives or compound libraries.
5. Investigate structure–activity relationships (SAR) to optimize binding interactions.
6. Validate ADMET predictions through experimental pharmacokinetic and toxicity studies.
7. Explore the biological impact of key SPI1 mutations in cellular or animal models.

## 4. Materials and Methods

### 4.1. Retrieval of nsSNPs from Public Database

The dataset of nonsynonymous single-nucleotide polymorphism (nsSNPs) for the *SPI1* gene was obtained from the Ensemble database [36] (Gene ID: ENSG00000066336). A missense filter was applied during the SNP query process to extract specifically missense variants. The SPI1 protein sequence was then obtained in FASTA format from the NCBI protein database under the accession number NP_003111.2. These missense variants were subsequently used for in silico functional and structural analysis.

### 4.2. Identification the Most Deleterious SNPs

To evaluate the potential functional or structural impact of each missense SNP in the *SPI1* gene, the PredictSNP server [37] (https://loschmidt.chemi.muni.cz/predictsnp1/, accessed on 1 May 2025) was utilized. This platform classified the given nsSNPs as normal, moderate or deleterious. To ensure more robust and accurate evaluation, this platform collects the outputs from multiple tools, including PhD-SNP, MAPP, PolyPhen-1, PolyPhen-2, SIFT and SNAP. All 825 missense nsSNPs retrieved from the Ensemble database were applied to the analysis using this meta-predictor.

### 4.3. Identification nsSNPs Within Conserved Domains of the SPI1 Gene

The InterPro tool (https://www.ebi.ac.uk/interpro/, accessed on 2 May 2025) [38] was used to identify the positions of nsSNPs within the conserved domains of SPI1. The InterPro incorporates multiple protein databases such as Gene3D, PIRSF, ProDom, Pfam, PRINTS, TIGRFAMs, and PROSITE, enabling robust and comprehensive recognition of protein motifs and domains. Additionally, information about motifs and domains was obtained from UniProt (ID: P17947).

### 4.4. Impact of Missense Variants on Structural Modeling of SPI1 Protein

To investigate the structural impact of the nine missense variations in the SPI1 gene, structural modeling was performed focusing on the C-terminal ETS DNA-binding domain. Due to the intrinsically disordered nature of the N-terminal region of SPI1, full-length modeling may introduce unreliable structural artifacts. Therefore, analysis was restricted to the ETS domain, for which experimentally resolved structures are available (e.g., PDB ID: 8EQG). Wild-type and mutant structures were generated using the SWISS-MODEL (https://swissmodel.expasy.org/interactive, accessed on 1 May 2025), and structural visualization and alignment were performed using UCSF Chimera (v1.19). The Cα–Cα distances between wild-type and mutant residues were calculated to assess local conformational changes induced by each mutation.

### 4.5. SPI1 Protein Stability Prediction

I-Mutant 3.0 and MUpro, two machine-based prediction servers, were utilized to evaluate the impact of *SPI1* missense variations on protein stability. I-Mutant (https://folding.biofold.org/cgi-bin/i-mutant2.0.cgi, accessed on 2 May 2025) was used by submitting the full length of the SPI1 protein sequence in FASTA format (NCBI ID: NP_003111.2), under conditions of 25 °C and PH at 7.0. The output includes the predicted direction of stability change, free energy change (ΔΔG), and Reliability Index (RI) [22]. MUpro (http://mupro.proteomics.ics.uci.edu/, accessed on 2 May 2025) was also utilized with the full-length SPI1 protein sequence. The evaluation using MUpro involved the stability change (increase or decrease) and a confidence score.

### 4.6. Identification of Post-Translational Modification Sites in SPI1

Post-translational modifications (PTMs) sites were predicted using the MusiteDeep server (https://www.musite.net/, accessed on 3 May 2025) [39]. The full-length SPI1 protein sequence was obtained from NCBI (ID: NP_003111.2) and was submitted for the analysis. The prediction was performed for ubiquitination, phosphorylation, acetylation and methylation.

### 4.7. Protein Interaction Network for SPI1

To explore the interaction partners and biological role of SPI1 in the hematopoietic process, the STRING v12.0 database (https://string-db.org/cgi/input?sessionId=bNQnOMcoObr8&input_page_show_search=on, accessed on 3 May 2025) was used to construct a protein–protein (PPI) network. The full wild-type SPI1 protein sequence was submitted to the STRING to generate interaction outputs. Furthermore, the information of each protein within the network was retrieved from the STRING database which collects the information from different resources.

### 4.8. Sequence Conservation Analysis for SPI1

The full-length orthologous protein sequences of SPI1 for human, zebrafish and mouse were obtained in FASTA format and aligned using the MAFFT algorithm via Jalview v2.11.4.1. The evolutionary conservation of amino acids residues were assessed based on sequence similarity across the three species. Annotated mutation positions were labeled on the alignment. Furthermore, conservation bars and entropy plots were generated to evaluate the evolutionary conservation at each site.

### 4.9. Expression Analysis of SPI1

*SPI1* gene expression profile was examined using the GEPIA2 web tool (http://gepia2.cancer-pku.cn/#index, accessed on 4 May 2025). This database integrates RNA sequencing data from the Cancer Genome Atlas (TCGA) and Genotype-Tissue Expression (GTEx) project. A boxplot was generated to compare the expression of SPI1 in the tumor tissue in acute myeloid leukemia (LAML) against the normal tissue. The analysis was performed based on log_2_ (TPM + 1) normalization for more accurate statistical interpretation.

### 4.10. Molecular Docking Study

#### 4.10.1. Ligand Preparation

Prior to docking, cinnamic acid (SMILES: C1=CC=C(C=C1)/C=C/C(=O)O) was obtained in PubChem (PubChem, National Center for Biotechnology Information, Bethesda, MD, USA), and optimized using MMFF94 force field.

#### 4.10.2. Receptor Preparation

The domain structure of SPI1 ETS was formed with the addition of polar hydrogen and removal of water molecules (8EQG_modified.pdb). AutoDock Tools were used to assign Kollman charges.

#### 4.10.3. Docking Protocol

AutoDock Vina version 1.2.0 1.2.0 (SIB Swiss Institute of Bioinformatics, Lausanne, Switzerland) was used in molecular docking. The following parameters were used to set the grid box: center (6, −2, −9) AA; size 20 × 20 × 20 AA; exhaustiveness = 16. The binding affinity was obtained in kcal/mol. Minimum binding energy and favorable hydrogen bonding interactions in the best binding pose were picked. The best possible binding pose for each molecule was imagined using BIOVIA Discovery Studio Visualizer 2020 (Dassault Systèmes BIOVIA, San Diego, CA, USA) and PyMOL (version 3.1.7.2; Schrödinger, LLC, New York, NY, USA).Docking consistency was further validated using the SwissDock platform as an independent docking tool to assess the reproducibility of binding poses and affinity trends.

#### 4.10.4. Re-Docking Validation

The docking protocol was tested by a re-docking protocol, which involved removing the co-crystallized ligand from the crystal structure and re-docking the ligand back into the original binding site with the same docking parameters and configuration. For the docking simulations, the same grid box size and the same scoring parameters were used for all docking experiments and the docking software was AutoDock Vina 1.2.0.

#### 4.10.5. ADMET and Drug-Likeness Analysis

The absorption, distribution, metabolism, excretion and toxicity (ADMET) properties of the compound were predicted using the https://admet.ai.greenstonebio.com/ web server which is a graph-based signature system to estimate these parameters. This server was accessed on 1 February 2026.

## 5. Conclusions

This comprehensive bioinformatics analysis shows that some missense nsSNPs mutations within the SPI1 gene, particularly those situated in the conserved ETS domain, can markedly change SPI1 structure and stability, influencing its transcriptional regulatory roles. Prediction of the PTM site revealed that SPI1 may also be affected via post-translational regulatory mechanisms and not only by missense nsSNPs variants. The protein interaction analysis showed that SPI1, situated in the center of a network of hematopoietic transcription regulators and conservational prediction, demonstrated its functional indispensability across vertebrate species. The increase in SPI1 expression in AML tissues suggests the potential use of SPI1 as an early biomarker and therapeutic target. Overall, this study emphasizes the utility of computational analysis to investigate the molecular impact of missense nsSNPs variants on SPI1 structure, stability and function, providing a fundamental framework for experimental validation in the future.

Cinnamic acid has been shown to behave as a fragment in the PU.1 ETS domain, with an intermediate docking affinity and a restricted but structurally coherent profile of interaction. Its low molecular weight, desirable physicochemical characteristics and anticipated high oral absorption make it suitable as a lead-like scaffold rather than a potent inhibitor. The documented antioxidant, anti-inflammatory, antimicrobial, and antiproliferative properties of the compound are another strength in its biologic relevance. Altogether, cinnamic acid is a structurally simple yet pharmacologically versatile framework which could be used as starting point for rational derivatization and optimization to improve target specificity and therapeutic potential.

## Figures and Tables

**Table 1:** The nine deleterious sSNPs identified using seven computational prediction tools.

SNP ID	Mutation	PredictSNP	PolyPhen 1 and 2	MAPP, PHD-SNP, SIFT, SNAP
rs2142874766	G165D	Deleterious	Damaging	Deleterious
rs2095906645	I189F	Deleterious	Damaging	Deleterious
rs2095906547	H211P	Deleterious	Damaging	Deleterious
rs1595852995	R220L	Deleterious	Damaging	Deleterious
rs1338148822	A229V	Deleterious	Damaging	Deleterious
rs765064980	R230L	Deleterious	Damaging	Deleterious
rs2095906404	V241G	Deleterious	Damaging	Deleterious
rs1225370403	K247Q	Deleterious	Damaging	Deleterious
rs748655252	R259C	Deleterious	Damaging	Deleterious

**Table 2:** Computational prediction of SPI1 missense mutations on protein stability using I- Mutant and MUpro.

SNP ID	Mutation	I-Mutant Stability Change	I-Mutant ΔΔG (kcal/mol)	Reliability Index	MUpro Stability	MUpro ΔΔG (kcal/mol)
rs2142874766	G165D	Decrease	−0.55	2	Decrease	−0.341122
rs2095906645	I189F	Decrease	−1.5	8	Decrease	−1.524400
rs2095906547	H211P	Increase	−0.70	3	Decrease	−1.495726
rs1595852995	R220L	Decrease	−0.47	5	Decrease	−0.339015
rs1338148822	A229V	Decrease	−0.90	4	Decrease	−0.328434
rs765064980	R230L	Decrease	−0.23	4	Increase	0.156723
rs2095906404	V241G	Decrease	−3.35	9	Decrease	−2.414506
rs1225370403	K247Q	Increase	0.09	3	Decrease	−0.641548
rs748655252	R259C	Decrease	−0.78	5	Decrease	−0.691126

**Table 3:** Predicted post-translational modification sites in SPI1 protein identified by MusiteDeep.

Amino Acid	Position	Post-Translation Modification Site	Confidence Score
T	164	Phosphorylation	0.192
S	166	Phosphorylation	0.379
S	188	Phosphorylation	0.177
R	212	Methylation	0.168
N	219	Glycosylation	0.033
R	220	Methylation	0.154
K	221	Ubiquitination	0.336
R	230	Methylation	0.12
K	242	Acetylation	0.413
K	246	Acetylation	0.206
K	247	Acetylation	0.41
R	259	Methylation	0.93

**Table 4:** Consolidated ADMET profile of cinnamic acid.

Category	Parameter	Predicted Value	Interpretation
Physicochemical	Molecular Weight	148.16 Da	Low MW; favors permeability
	LogP	1.78	Moderate lipophilicity
	H-Bond Acceptors	1	Within Lipinski limits
	H-Bond Donors	1	Within Lipinski limits
	Lipinski Rule	4/4	Fully compliant
	QED	0.65	Acceptable drug-likeness
	TPSA	37.30 Å^2^	Supports good absorption and BBB penetration
Absorption	Human Intestinal Absorption	0.99	Excellent oral absorption
	Oral Bioavailability	0.91	High systemic availability
	Aqueous Solubility	−2.36 log(mol/L)	Moderate solubility
	Cell Permeability	−4.58 log(10^−6^ cm/s)	Favorable membrane diffusion
	P-gp Inhibition	0.004	Minimal efflux interaction
Distribution	BBB Penetration	0.77	Moderate CNS permeability
	Plasma Protein Binding	92.47%	High binding affinity
	Volume of Distribution	4.61 L/kg	Extensive tissue distribution
Metabolism	CYP1A2 Inhibition	0.04	Low inhibition risk
	CYP2C19 Inhibition	0.03	Low inhibition risk
	CYP2C9 Inhibition	0.04	Low inhibition risk
	CYP2D6 Inhibition	0.01	Very low inhibition risk
	CYP3A4 Inhibition	0.0003	Negligible inhibition
	CYP2C9 Substrate	0.44	Possible metabolic substrate
Excretion	Half-Life	0 h (predicted)	Likely short systemic persistence
	Microsomal Clearance	3.22 µL/min/mg	Moderate metabolic clearance
Toxicity	hERG Blocking	0.01	Low cardiotoxicity risk
	Mutagenicity	0.02	Low genotoxic risk
	Drug-Induced Liver Injury	0.91	High predicted hepatotoxicity risk
	Carcinogenicity	0.24	Moderate risk
	Acute Toxicity (LD50)	1.58 log(1/(mol/kg))	Low acute toxicity
	Skin Reaction	0.61	Moderate irritation potential

**Table 5:** Swiss target prediction profile of cinnamic acid.

Rank	Target (Common Name)	Gene	UniProt ID	Target Class	Probability
1	Hydroxycarboxylic acid receptor 2	HCAR2	Q8TDS4	Family A GPCR	**0.8870**
2	Aldose reductase	AKR1B1	P15121	Oxidoreductase (Enzyme)	0.1012
3	Toll-like receptor 4 (by homology)	TLR4	O00206	Toll-like receptor	0.0918
4	Carbonic anhydrase II	CA2	P00918	Lyase	0.0823
5	Carbonic anhydrase I	CA1	P00915	Lyase	0.0823
6	Estrogen receptor beta	ESR2	Q92731	Nuclear receptor	0.0823
7	Monocarboxylate transporter 1 (by homology)	SLC16A1	P53985	Electrochemical transporter	0.0823
8	Carbonic anhydrase IX	CA9	Q16790	Lyase	0.0727
9	Transient receptor potential cation channel A1	TRPA1	O75762	Ion channel	0.0727
10	Matrix metalloproteinase 9	MMP9	P14780	Protease	0.0535
11	Matrix metalloproteinase 2	MMP2	P08253	Protease	0.0535
12	Protein-tyrosine phosphatase 1B	PTPN1	P18031	Phosphatase	0.0535
13	Epidermal growth factor receptor	EGFR	P00533	Kinase	0.0439

## Data Availability

The original contributions presented in this study are included in the article. Further inquiries can be directed to the corresponding author.
